# Supplementary material for: Effect of telomere shortening on disease progression in patients with inflammatory bowel disease: A systematic review and meta-analysis protocol
Source: PLoS One. 2024 Oct 16;19(10):e0311662. doi: 10.1371/journal.pone.0311662 (PMC11482682; doi:10.1371/journal.pone.0311662)
Supplement: S1 File — (DOC) [file pone.0311662.s001.doc]

**PRISMA-P (Preferred Reporting Items for Systematic review and Meta-Analysis Protocols) 2015 checklist: recommended items to address in a systematic review protocol***

| Section and topic | Item No | Checklist item |
| --- | --- | --- |
| ADMINISTRATIVE INFORMATION | | |
| Title: |  |  |
| Identification | 1a | Effect of telomere shortening on disease progression in patients with inflammatory bowel disease: a systematic review and meta-analysis protocol |
| Update | 1b |  |
| Registration | 2 | CRD42024501171 |
| Authors: |  |  |
| Contact | 3a | Yifan Zhang, [Doc_zhang_01@163.com](mailto:Doc_zhang_01@163.com), School of Traditional Chinese Medicine, Beijing University of Chinese Medicine;  Ze Ma, [837223303@qq.com](mailto:837223303@qq.com), Graduate School, Tianjin University of Traditional Chinese Medicine;  Liang Kang, [247571755@qq.com](mailto:247571755@qq.com), Department of Cardiovascular, Guangzhou University of Chinese Medicine;  Liu Yang, [985618688@qq.com](mailto:985618688@qq.com), Department of Gastroenterology, Tianjin Academy of Traditional Chinese Medicine Affiliated Hospital |
| Contributions | 3b | Conceptualization: Yifan Zhang, Ze Ma.  Formal analysis: Yifan Zhang, Ling Kang.  Funding acquisition: Liu Yang.  Investigation: Ze Ma, Liang Kang.  Methodology: Yifan Zhang, Ze Ma.  Supervision: Liu Yang.  Validation: Ze Ma.  Writing-original draft: Yifan Zhang  Writing-review & editing: Yifan Zhang, Ze Ma, Liu Yang. |
| Amendments | 4 | not applicable |
| Support: |  |  |
| Sources | 5a | not applicable |
| Sponsor | 5b | not applicable |
| Role of sponsor or funder | 5c | not applicable |
| INTRODUCTION | | |
| Rationale | 6 | Inflammatory bowel disease (IBD) remains a major public health challenge worldwide. IBD consists of two main disease subtypes: Ulcerative colitis (UC) and Crohn's disease (CD). UC is a chronic non-specific inflammatory bowel disease that begins in the rectal mucosa and continues to extend, while CD is a chronic non-specific granulomatous inflammation that tends to occur in the distal ileum and cecum. Recent epidemiological surveys have shown a significant growth in the incidence of IBD in the Americas, Europe, Asia, and Africa [1]. Specifically, more than 1.5 million and 2 million people in North America and Europe are suffering from the IBD [2], and Japan’s national registry system has recorded an increase in the prevalence of UC and CD (172.9 cases/105/year and 55.6/105/year, respectively) [3]. IBD is predominantly targeted at young adults [4], but the continued rise in prevalence is partly influenced by an aging population, with 10 to 15 percent of IBD diagnoses occurring in individuals over 50 years of age [5].  IBD patients is characterized by recurrent episodes, gradually shortening of the interval between attacks, and progressively severe disease. These features further augment the physical and mental challenges of patients. For IBD, while momentous theoretical advances have been made by scholars, the etiology of IBD remains largely unclear. Many studies have highlighted the critical role of the environment, genetic factors, the immune system, and the microbiota [6,7]. It is worth noting that in the long-term course of the IBD, multiple etiological interactions drive the dysregulation of biological processes [8].  After the etiology-driven occurrence of IBD, a state of chronic low-grade inflammation permeates the disease. Chronic low-grade inflammation is a hallmark of aging and is achieved by affecting the length and function of telomeres [9,10]. Telomere shortening, in turn, often translates into disease and aging in humans [11]. Therefore, telomere length, as a cellular biomarker of aging, can predict physical health and longevity. The available evidence suggests the presence of short telomeres in the colonic mucosa in IBD patients [12-14]. Although telomere length reduces with age in the normal colon, the rate of telomere shortening is significantly faster in IBD patients. Similarly, some studies suggest that telomere shortening due to telomeropathies serves as one of the underlying factors for the early onset of IBD. And further promote disease progression, resulting in the development of colon cancer [15].  The above research indicates an association between telomere shortening and IBD disease development, However, there is controversy as to whether telomere shortening precipitates disease progression or disease progression causes telomere shortening. There is also a shortage of systematic reviews and data synthesis to explain the connection between telomere shortening and disease advancement in individuals with IBD. Therefore, a systematic review of qualitative and quantitative meta-analyses will be done here to seek the relationship both illness progression in IBD patients and telomere shortening for future research purposes. |
| Objectives | 7 | The objective of this review is to examine and synthesize the literature on the association of telomere shortening with disease progression in people with IBD.  Specifically, we aim to:  1.Summarize the literature on the relationship between telomere shortening and disease progression in patients with IBD (primary outcomes: to determine the correlation between disease progression and telomere shortening and the causal relationship between telomere shortening and disease progression; secondary outcome: relationship between disease duration and telomere shortening).  2.If possible, pool studies together to conduct a meta-analysis.  3.Conduct subgroup analyses based on disease subtype, age, country, and treatment of IBD (receiving treatment versus not receiving treatment) and comorbidity.  P: Adult participants (age > 18 years) with a clinical diagnosis or colonoscopically confirmed IBD (including only the UC and CD subtypes)  I (E): Inflammatory bowel disease  C: Normal population  O: the relationship between telomere shortening and disease progression in patients with IBD |
| METHODS | | |
| Eligibility criteria | 8 | The investigations will consist of cross-sectional observational, case-control, and cohort studies examining the relationship both disease advancement in IBD patients and telomere shortening. Adult participants (age > 18 years) with a clinical diagnosis or colonoscopically confirmed IBD (including only the UC and CD subtypes) will be included in the study. The control group consisted of adults without IBD. The primary outcome measure will determine the association between disease progression and telomere shortening, defined as the shortening of telomeres in patients with IBD due to each cell division and cellular stress compared to the normal population. As the disease progresses, the degree of telomere shortening is further determined and evaluated by quantitative polymerase chain reaction [19]. Disease progression refers to the progression of the disease from the diagnosis of IBD and is expressed in grades of mild, moderate, and severe. Assessed by clinical symptoms (e.g., frequency of bowel movements, abdominal pain), laboratory tests (e.g., erythrocyte sedimentation rate, occult blood score), and pathologic analysis [20]. Evaluations will be conducted in hospital and community settings, encompassing both inpatient and outpatient environments. All variables such as gender, nationality, language, research population, and study design were not limited.  Excluded studies include those that are incomplete (such as ongoing trials and preliminary results), involve animal subjects, or are in vitro studies. In the event of repeated publication of studies with the same population, preference will be given to the report with the largest sample size. In addition, conference proceedings or abstracts, reviews, editorials, and review papers will not be considered. |
| Information sources | 9 | From inception until December 31, 2023, we will conduct a comprehensive search of the electronic database, and there are no language restrictions in the search strategy. We will search the following databases: MEDLINE/PubMed, Embase, Web of Science, China National Knowledge Infrastructure (CNKI), China Scientific Journals Database (VIP), Wanfang Database (Wanfang), China Biomedical Literature Database (CMB), Cochrane Library, Cochran Clinical Trials Registry, and the World Health Organization International Clinical Trials Registry Platform. In addition, to identify relevant grey literature, we will search databases of papers and search the reference list of included studies for additional articles and resources. If information from the included studies is missing, we will contact the authors to obtain that information and record any correspondence. |
| Search strategy | 10 | The search strategy includes the necessary keyword fields, using MeSH terms and free word searches. The search included “inflammatory bowel disease”, “bowel disease, inflammatory”, “Ulcerative colitis”, “Crohn’s disease”, “telomere shortening”, “telomere shortenings”, “shortening, telomere”, “shortenings, telomere”, “disease progression”, “progression, disease”, “clinical progression”, “progression, clinical”, “disease exacerbation”, “exacerbation, disease”. In addition, we present the search strategy for one of the databases (Detailed search strategies are provided in the manuscript.) and will include the full search strategy table for all databases in a comprehensive review. These databases will be searchable from the beginning to the present, with no language or demographic restrictions, and will be limited to human research. |
| Study records: |  |  |
| Data management | 11a | The information will be transferred into Review Manager 5.3 for data management. |
| Selection process | 11b | All extracted literature was reviewed in three stages: title and abstract screening, full-text screening, and data extraction. This process will be carried out in duplicate. Any disagreements in the included studies will be resolved by the reviewers through rigorous discussion, and any remaining disagreements will be addressed and resolved by a third. |
| Data collection process | 11c | The data extraction form will be created in duplicate. The following items will be collected for each article included for data extraction: author, year, country, gender, age (including the true age of patients in the experimental and control groups and the age of onset of the patients in the experimental group), sample size (including the total sample size and the number of people assigned to the experimental and control groups), duration of disease, disease subtype (UC/CD), disease stage (mild, moderate, or severe), drug treatment, comorbidity, and telomere length (telomere shortening). If information from the included studies is missing, we will contact the authors to obtain that information and record any correspondence. Eventually, if this information is not obtained, the data will be excluded from the analysis. |
| Data items | 12 | The following items will be collected for each article included for data extraction: author, year, country, gender, age (including the true age of patients in the experimental and control groups and the age of onset of the patients in the experimental group), sample size (including the total sample size and the number of people with IBD and the normal population), duration of disease, disease subtype (UC/CD), disease stage (mild, moderate, or severe), drug treatment, comorbidity, and telomere length (telomere shortening). |
| Outcomes and prioritization | 13 | The primary objective of this review will be to determine the association between telomere shortening and disease progression in people with IBD. Secondary outcomes for this review include the relationship between disease duration and telomere shortening. The duration of the disease, or the number of years of disease, has a cumulative effect on telomere shortening. |
| Risk of bias in individual studies | 14 | Using the proper instruments following the study design, and the risk of bias in each study that is part of the data extraction phase will be evaluated. These evaluations will also be finished by two reviewers, respectively. We will apply the Newcastle-Ottawa Scale (NOS) to assess the risk of bias [21]. If possible, we will perform sensitivity analyses for papers with low scores at risk of bias. |
| Data synthesis | 15a | We will only pool studies with a low or moderate risk of bias. |
|  | 15b | We will use the χ2 test and I^2^ statistic test to assess the heterogeneity. If the results meet the I^2^ value is <50% and the p>0.10, we pooled the data using a fixed-effect model. Otherwise, a random-effects model will be used. |
|  | 15c | After analysing using the NOS risk of bias tool, we performed sensitivity analyses for papers with low scores of bias. If the number of studies is sufficient, and there is substantial heterogeneity between studies, subgroup analyses will be performed. Expected sources of heterogeneity include disease subtype, drug use, age, country, and comorbidity and if significant heterogeneity is found, this will be flagged and subgroup analysed for this. |
|  | 15d | If a meta-analysis was not possible (i.e. studies were small and heterogeneous were high), we performed a narrative synthesis of the included papers to sum up the results of the included studies. |
| Meta-bias(es) | 16 | A funnel plot will be used to analyse for potential reporting bias when the number of studies exceeds 10 in the meta-analysis. Heterogeneity of the funnel plot will be determined by Egger's test. In addition, p<0.05 would imply an important reporting bias. |
| Confidence in cumulative evidence | 17 | Two reviewers will assess the strength of the evidence using the Grading of Recommendations Assessment, Development and Evaluations (GRADE) system. The quality of the evidence will be assessed for the following items: risk of bias, publication bias, inconsistency, indirectness, and imprecision. |

*** It is strongly recommended that this checklist be read in conjunction with the PRISMA-P Explanation and Elaboration (cite when available) for important clarification on the items. Amendments to a review protocol should be tracked and dated. The copyright for PRISMA-P (including checklist) is held by the PRISMA-P Group and is distributed under a Creative Commons Attribution Licence 4.0.**

*From: Shamseer L, Moher D, Clarke M, Ghersi D, Liberati A, Petticrew M, Shekelle P, Stewart L, PRISMA-P Group. Preferred reporting items for systematic review and meta-analysis protocols (PRISMA-P) 2015: elaboration and explanation. BMJ. 2015 Jan 2;349(jan02 1):g7647.*
